# Disability trajectories by progression independent of relapse activity status differ in pediatric, adult and late-onset multiple sclerosis

**DOI:** 10.1007/s00415-024-12638-0

**Published:** 2024-08-23

**Authors:** Marta Simone, Giuseppe Lucisano, Tommaso Guerra, Damiano Paolicelli, Maria A. Rocca, Vincenzo Brescia Morra, Francesco Patti, Pietro Annovazzi, Claudio Gasperini, Giovanna De Luca, Diana Ferraro, Lucia Margari, Franco Granella, Carlo Pozzilli, Silvia Romano, Paola Perini, Roberto Bergamaschi, Maria Gabriella Coniglio, Giacomo Lus, Marika Vianello, Alessandra Lugaresi, Emilio Portaccio, Massimo Filippi, Maria Pia Amato, Pietro Iaffaldano, the Italian Multiple Sclerosis Register

**Affiliations:** 1 Child Neuropsychiatry Unit, Department of Precision and Regenerative Medicine, Jonic Area University of Bari “Aldo Moro”, Bari, Italy; 2 CORESEARCH, Pescara, Italy; 3 Department of Translational Biomedicine and Neurosciences–DiBraiN, University “Aldo Moro” Bari, Piazza Giulio Cesare 11, 70124 Bari, Italy; 4 Dipartimento di Neurologia, Neurofisiologia e Neuroriabilitazione, San Raffaele Scientific Institute, Vita-Salute San Raffaele University, Milan, Italy; 5 Department of Neuroscience (NSRO), Multiple Sclerosis Clinical Care and Research Center, Federico II University, Naples, Italy; 6 Dipartimento di Scienze Mediche e Chirurgiche e Tecnologie Avanzate, GF Ingrassia, Sez. Neuroscienze, Centro Sclerosi Multipla, Università di Catania, Catania, Italy; 7 Neuroimmunology Unit - Multiple Sclerosis Centre ASST Valle Olona, Gallarate Hospital, Gallarate, Italy; 8 Department of Neurosciences, S.Camillo Forlanini Hospital, Rome, Italy; 9 Centro Sclerosi Multipla, Clinica Neurologica, Policlinico SS. Annunziata, Chieti, Italy; 10 Azienda Ospedaliera Universitaria di Modena/OCB, UO Neurologia, Milano, Italy; 11 Unit of Neurosciences, Department of Medicine and Surgery, University of Parma, Parma, Italy; 12 Department of Human Neuroscience, Multiple Sclerosis Center, S. Andrea Hospital, Rome, Italy; 13 Department of Neurosciences, Mental Health and Sensory Organs, Centre for Experimental Neurological Therapies (CENTERS), Sapienza University of Rome, Rome, Italy; 14 Department of Neurosciences, Multiple Sclerosis Centre-Veneto Region (CeSMuV), University Hospital of Padua, Padua, Italy; 15 IRCCS Mondino Foundation, Pavia, Italy; 16 Center for Multiple Sclerosis, Hospital ASM “Madonna delle Grazie”, 75100 Matera, Italy; 17 Multiple Sclerosis Center, II Division of Neurology, Department of Clinical and Experimental Medicine, Second University of Naples, Naples, Italy; 18 Unit of Neurology, Cà Foncello Hospital, Treviso, Italy; 19 IRCCS Istituto Scienze Neurologiche di Bologna, Bologna, Italy; 20 Dipartimento di Scienze Biomediche e Neuromotorie, Università di Bologna, Bologna, Italy; 21 Department NEUROFARBA, University of Florence, Florence, Italy; 22 IRCCS Fondazione Don Carlo Gnocchi, Florence, Italy

**Keywords:** PIRA, Disability trajectories, POMS, AOMS, LOMS

## Abstract

**Background:**

To compare Expanded Disability Status Scale (EDSS) trajectories over time between Multiple Sclerosis (MS) groups with pediatric (POMS), adult (AOMS) and late (LOMS) onset, and between patients with and without progression independent of relapse activity (PIRA).

**Methods:**

Patients with a first visit within 1 year from onset, ≥ 5-year follow-up and ≥ 1 visit every 6 months were selected from the Italian MS Register. Adjusted disability trajectories were assessed by longitudinal models for repeated measures. Comparisons between groups and between patients with and without PIRA in subgroups were performed by evaluating the yearly differences of mean EDSS score changes versus baseline (delta-EDSS).

A first CDA event was defined as a 6-months confirmed disability increase from study baseline, measured by EDSS (increase ≥ 1.5 points with baseline EDSS = 0; ≥ 1.0 with baseline EDSS score ≤ 5.0 and ≥ 0.5 point with baseline EDSS > 5.5).

PIRA was defined as a CDA event occurring more than 90 days after and more than 30 days before the onset of a relapse.

**Results:**

3777 MS patients (268 POMS, 3282 AOMS, 227 LOMS) were included. The slope of disability trajectories significantly diverged in AOMS vs POMS starting from the second year of follow-up (Year 2: delta2-EDSS 0.18 (0.05; 0.31), p = 0.0054) and then mean delta2-EDSS gradually increased up to 0.23 (0.07; 0.39, p = 0.004) at year 5. Patients with PIRA had significant (p < 0.0001) steeper increase in EDSS scores than those without PIRA in all groups, although in POMS, the disability trajectories began to diverge later and at a lesser extent with delta-EDSS score of 0.48 vs 0.83 in AOMS and 1.57 in LOMS, at 3 years after the first PIRA.

**Conclusions:**

Age is relevant in determining disability progression in MS. POMS shows a less steep increase in EDSS scores over time than older patients. The effect of PIRA in accelerating EDSS progression is less pronounced in POMS than in AOMS and LOMS.

## Introduction

Growing knowledge about multiple sclerosis (MS) allowed to delineate the disease course as a *continuum*, whereby neurodegeneration and neuroinflammation constitute the pathological substrate of a progressive disability [1–3]. Phenotypic differences in clinical disability progression across patients and within individual patients over time results from a combination of several mechanisms, including patient-specific factors, such as age, sex, environmental and genetic factors [4]. Age is one of the major patient-specific factors influencing the disease course [5].

MS onset is typically in adults (adult onset – AOMS) between the ages of 20 and 40 years, while pediatric (POMS) and late-onset (LOMS) forms are less frequent but increasingly studied [6, 7]. In relapsing–remitting MS (RRMS), it is becoming evident that the irreversible disability accrual is the result of relapse-associated worsening (RAW) combined with progression independent of relapse activity (PIRA) throughout the disease course. Both randomised clinical trials and observational studies demonstrated that steady PIRA is the main driver of the accumulation of disability across the full spectrum of MS phenotypes since the earliest phases of the disease [8–12]. A recent study of our group [13] showed PIRA events accounted for 40% of the first confirmed disability accrual (CDA) in POMS, for 84.20% in AOMS and for 90.45% in LOMS, and an early PIRA, occurring within the first five years of the disease, was present in 41.0% of POMS, 45.0% of AOMS and 54.4% of LOMS subjects. Patients with AOMS, presenting with PIRA after a first demyelinating event, have been demonstrated to have an unfavourable long-term prognosis with a significant steeper increase in expanded disability status (EDSS) scores over time than those without PIRA, especially if it occurs early in the disease course [14]. The association of a first PIRA event with disability trajectories over time in POMS and LOMS patients has not been investigated so far.

Registry-based studies have proven to be a valuable guide in mapping the disability trajectories of MS patients of all ages, combining biological, clinical, and therapeutic data [15–17].

Following this research path, in a large real life-cohort of disease modifying drugs (DMT) treated MS patients from the Italian MS and Related Disorders Register (I-MS&RD) [18], we assessed and compared disability trajectories by PIRA status over time in three subgroups of patients stratified by age at clinical onset, ≤ 18 (POMS), 19–49 (AOMS), > 49 (LOMS) years.

## Materials and methods

### Data extraction

This is a retrospective observational cohort study based on prospectively collected clinical data from the I-MS&RD. Data extraction was executed in September 2021. The I-MS&RD was approved by the ethical committee at the “Azienda Ospedaliero – Universitaria – Policlinico of Bari” (Study REGISTRO SM001 – approved on 8 July 2016) and by local ethics committees in all participating centres. Patients signed an informed consent that allows us to use their clinical data for research purposes. According to the Registry rules, on 5 February 2018, the Scientific Committee of the I-MS&RD granted the approval to conduct this project and extract and use the registry data.

We selected RRMS patients with a follow-up of at least 5 years, a first visit within one year after disease onset and EDSS scores regularly collected every 6 months.

EDSS scores were obtained by certified EDSS raters at all MS centers.

The following variables were included in the dataset: date of birth, sex, date of disease onset, dates of relapses, dates of EDSS evaluations, start- and end-dates of all the administered DMTs.

DMTs were classified based on moderate efficacy (ME: interferon beta products, glatiramer acetate, teriflunomide, dimethyl fumarate, azathioprine.) and high efficacy (HE: natalizumab, fingolimod, mitoxantrone, rituximab, cladribine, cyclophosphamide.)

### Statistical Analysis

For the entire cohort, baseline characteristics have been calculated as medians with interquartile ranges (IQR) and minimum maximum for continuous variables, and categorical variables have been presented as frequencies (proportions).

The cohort was stratified into three subgroups based on age at clinical onset: ≤ 18 (POMS), 19–49 (AOMS), > 49 (LOMS) years.

Between groups comparisons were performed by using the Student’s t test (for continuous variables normally distributed) or the Mann–Whitney test (for continuous variables not normally distributed), the ANOVA test (for continuous variables) or the chi-square test (for categorical variables).

The disability trajectories in the three subgroups were evaluated by applying a longitudinal mixed model for repeated measures (LMMRM) with an autoregressive unstructured variance–covariance structure, which included the following covariates: sex, proportion of follow-up time spent on active DMT exposure (pDMTs), relapses, time to first DMT and time to first visit. LMMRM with an autoregressive correlation-type matrix makes an assumption of missing at random and accounts for both missingness at random and potential correlation within participants, because it allows evaluation of all individuals, including participants with incomplete data [19].

The adjusted evolution over time of the disability accumulation was assessed by calculating the mean annual estimated EDSS changes compared to baseline estimated EDSS values (delta-EDSS). The comparisons between the 3 groups were performed by evaluating the yearly differences of the delta-EDSS (delta_2_-EDSS) in 3 pairwise comparisons (POMS vs AOMS; POMS vs LOMS; AOMS vs LOMS).

**Fig. 1:**
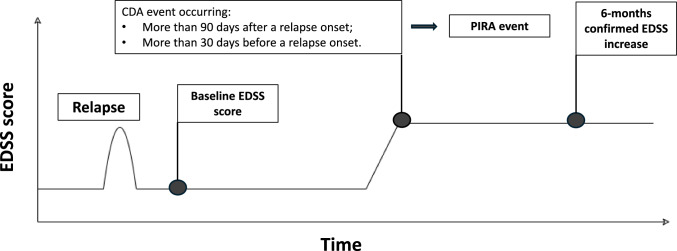
Visual representation of PIRA definition

A first CDA event was defined (Fig. 1) as a 6-months confirmed disability increase from study baseline, measured by EDSS (increase ≥ 1.5 points with baseline EDSS = 0; ≥ 1.0 with baseline EDSS score ≤ 5.0 and ≥ 0.5 point with baseline EDSS > 5.5).

Date of CDA was assigned at the first EDSS when an increase was registered. PIRA was defined (Fig. 1) as a CDA event occurring  more than 90 days after and more than 30 days before the onset of a relapse [20].

Then, we evaluated EDSS trajectories over time for patients stratified by the occurrence of PIRA events in the 3 groups.

The comparison between the PIRA and non-PIRA EDSS trajectories was performed by evaluating the yearly differences of the delta-EDSS (delta_3_-EDSS) in 3 subgroups since the median time of the occurrence of the first PIRA event.

A p value < 0.05 was considered statistically significant. All statistical tests were two-tailed. Analyses were performed using R version 3.2.0.

## Results

Longitudinal clinical data of more than 70,000 patients from 120 MS centres were available in the I-MS&RD at the time of data extraction. After applying the restrictive inclusion and exclusion criteria we retrieved a cohort of 3777 MS patients. The demographic and clinical characteristics of the entire cohort and of the three subgroups of patients stratified by age at clinical onset are shown in Table 1**.** The POMS group included 268 patients with a median (interquartile range – IQR) age at clinical onset of 16.00 (14.50–17.00) years. The AOMS group was composed by 3282 patients with a median (IQR) age at clinical onset of 31.00 (25.00–38.00) years, whereas the LOMS group included 227 patients with a median (IQR) age at clinical onset of 53.00 (51.00–56.00) years. There were significant (p = 0.05) differences among the three groups in terms of sex, being male sex more frequent in LOMS (86 subjects, 37.89%) in comparison to AOMS (1079 subjects, 32.88%) and POMS (74 subjects, 27.61%), and in terms of clinical disease activity, with a significantly (p = 0.03) lower proportion of LOMS (61 patients, 26.87%) reporting at least 1 relapse in the period between disease onset and the first visit in comparison to AOMS (1088 patients, 33.15%) and POMS (105 patients, 39.17%). The baseline median EDSS score was significantly (p < 0.0001) higher in LOMS (2.00, 1.50–2.50) in comparison to POMS and AOMS (1.50, 1.00–2.00).

**Table 1:** Demographic and clinical characteristics of the entire RMS cohort and the three subgroups stratified by age at onset

Variable	Overall	POMS N = 268	AOMS N = 3282	LOMS N = 227	P value
Age at onset, years, median (IQR), [min–max]	31.00, (24.00–39.00), [1.00–73.00]	16.00, (14.50–17.00), [1.00–18.00]	31.00, (25.00–38.00), [18.10–49.00]	53.00, (51.00–56.00), [49.10–73.00]	< 0.0001
Age at first prescription, years, median (IQR), min–max	31.85, (25.80–39–80), [1.00–74.00]	16.40, (14.90–17.80), [1.00–23.20]	32.00, (26.10–38.80), [16.40–61.80]	53.55, (51.50–57.10), [38.00–74.00]	< 0.0001
Male patients, n (%)	1239 (32.80)	74 (27.61)	1079 (32.88)	86 (37.89)	0.05
Disease duration, months	3.40 (1.30–6.90)	3.70 (1.40–7.30)	3.40 (1.30–6.80)	3.10 (1.20–6.90)	0.51
Type of clinical onset, n (%)					
Monofocal	3144 (83.24)	227 (84.70)	2727 (83.09)	190 (83.70)	0.73
Multifocal	539 (14.27)	36 (13.43)	474 (14.44)	29 (12.78)	
Number of relapses between the disease onset and the first visit, classes, n (%)					
0	2523 (66.80)	163 (60.82)	2194 (66.85)	166 (73.13)	0.03
1	975 (25.81)	78 (29.10)	845 (25.75)	52 (22.91)	
≥ 2	279 (7.39)	27 (10.07)	243 (7.40)	9 (3.96)	
Baseline EDSS score, median (IQR)	1.50 (1.00–2.00)	1.50 (1.00–2.00)	1.50 (1.00–2.00)	2.00 (1.50–2.50)	< 0.0001
At least 1 DMT prescription, during the 5 year follow-up n (%)	3654 (96.74)	262 (97.76)	3181 (96.92)	211 (92.95)	0.003
Total DMT exposure duration, years, median (IQR)	4.37 (1.93–4.95)	4.09 (0.78–4.99)	4.37 (1.93–4.95)	4.53 (3.19–4.91)	0.24
Time from disease onset to first DMT start, months	6.90 (3.40–11.50)	6.85 (3.50–11.25)	6.90 (3.40–11.50)	7.00 (3.00–12.00)	0.91
Time from disease onset to first DMT start stratified in < 6 months and ≥ 6 months, n (%)					
< 6 months	1704 (45.12)	119 (44.40)	1486 (45.28)	99 (43.61)	0.86
≥ 6 months	2073 (54.88)	149 (55.60)	1796 (54.72)	128 (56.39)	
First DMT classified as ME*or HE**, n (%)					
ME DMT	3233 (88.48)	229 (87.40)	2813 (88.43)	191 (90.52)	0.56
HE DMT	421 (11.52)	33 (12.60)	368 (11.57)	20 (9.48)	
Proportion of patients who were exposed to a vertical switch during the follow-up, n (%)	1323 (39.46)	146 (63.20)	1135 (38.80)	42 (21.32)	< 0.0001

*RMS* relapsing multiple sclerosis, *POMS* pediatric onset MS, *AOMS* adult onset MS, *LOMS* late onset MS, *EDSS* expanded disability status scale, *DMT* disease modifying therapy; *ME* moderate efficacy, *HE* high efficacy

*ME DMT group is composed by: interferon beta products, glatiramer acetate, teriflunomide, dimethyl fumarate, azathioprine

**HE DMT groups is composed by: natalizumab, fingolimod, mitoxantrone, rituximab, cladribine, cyclophosphamide

No differences were found among the groups referring to the DMT exposure, both in terms of total exposure time and proportion of follow-up time spent on pDMT. Moreover, the proportion of patients starting with a moderate efficacy or with a high efficacy DMT did not differ among the groups. During the follow-up a significant (p < 0.0001) higher proportion of POMS (146 subjects, 63.20%), switched to more effective therapies in comparison to AOMS (1135 subjects, 38.80%) and LOMS (42 subjects, 21.32%). (Table 1).

The estimated mean baseline EDSS (95% CI) value was 1.58 (1.45–1.71) in POMS, 1.63 (1.59–1.67) in AOMS and 2.03 (1.89–2.17) in LOMS.

**Fig. 2:**
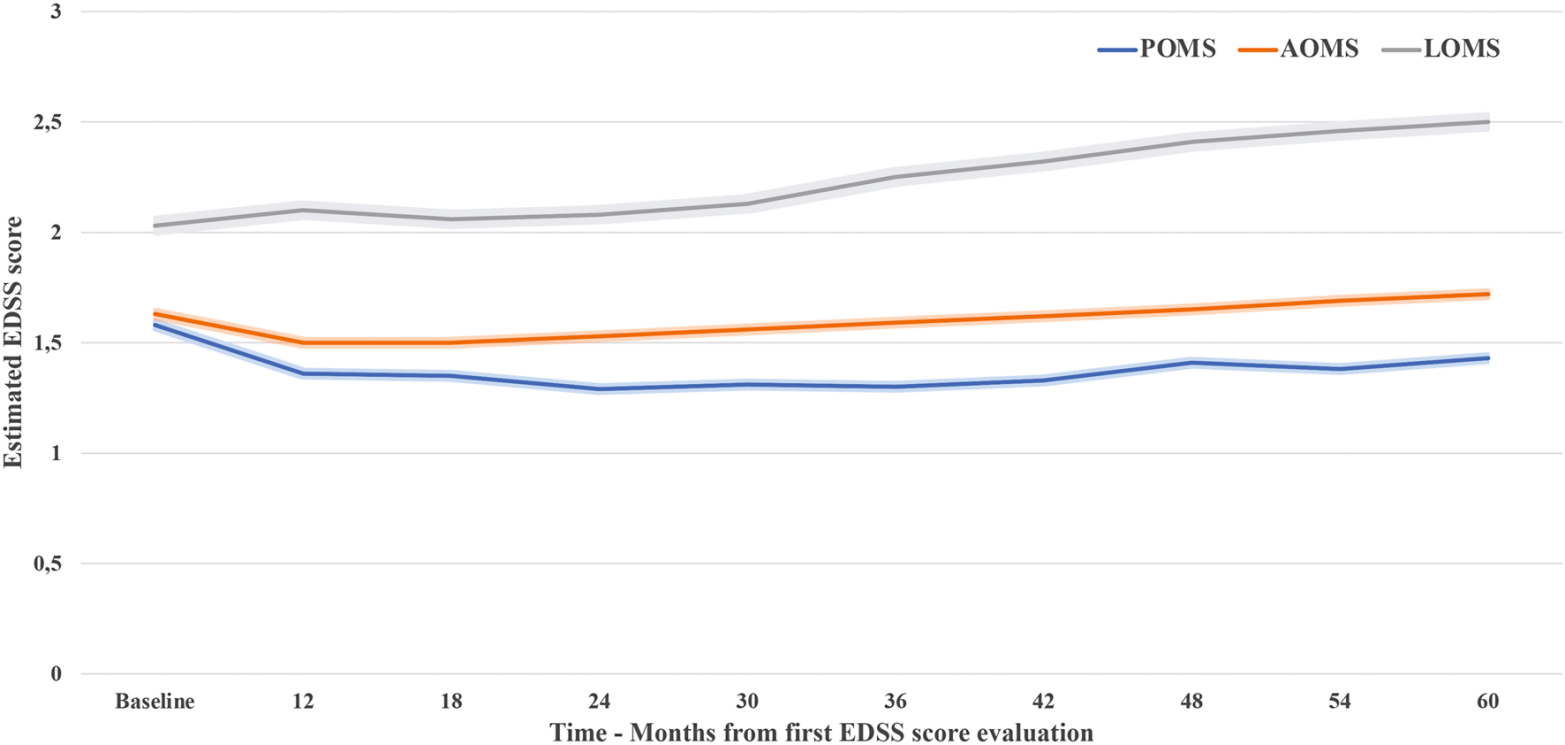
Disability trajectories based on the mean estimated delta-EDSS score with 95% CIs in POMS, AOMS and LOMS

The disability trajectories based on the mean estimated delta-EDSS score in POMS, AOMS and LOMS are shown in Fig. 2. POMS exhibited a flat disability trajectory, with a small, although significant, mean delta-EDSS reduction over time. The delta-EDSS trajectory of AOMS patients showed a significant decrease in the first 2 years of follow-up and then there was an increase which was significantly higher than the baseline value during the last year of follow-up.

Trajectory in LOMS followed a completely different trend compared to POMS and AOMS, presenting a baseline median EDSS score significantly higher in comparison to POMS and AOMS followed by a progressive increase of the EDSS score from the beginning of the observation.

The slope of disability trajectories significantly diverged in AOMS vs POMS starting from year 2 of follow-up (Year 2: delta_2_-EDSS 0.18 (0.05; 0.31), p = 0.0054) and then mean delta_2_-EDSS gradually increased up to 0.23 (0.07; 0.39, p = 0.004) at year 5 (Fig. 2 and Table 2**).**

**Table 2:** Comparison of annual estimated* mean delta_2_-EDSS scores (vs baseline) between POMS, AOMS and LOMS

Follow-up, years	AOMS vs POMS		LOMS vs POMS		LOMS vs AOMS	
	Estimated mean Delta_2_-EDSS scores (95% CI) vs baseline	P value	Estimated mean Delta_2_-EDSS scores (95% CI) vs baseline	P value	Estimated mean Delta_2_-EDSS scores (95% CI) vs baseline	P value
1 year	0.08 (− 0.04;0.20)	0.17	0.29 (0.12;0.46)	0.0008	0.21 (0.08;0.34)	0.002
2 years	0.18 (0.05;0.31)	0.005	0.34 (0.16;0.52)	0.0002	0.16 (0.02;0.30)	0.02
3 years	0.23 (0.09;0.37)	0.001	0.50 (0.30;0.70)	< 0.0001	0.27 (0.12;0.42)	0.0006
4 years	0.19 (0.04;0.34)	0.01	0.56 (0.35;0.77)	< 0.0001	0.37 (0.21;0.53)	< 0.0001
5 years	0.23 (0.07;0.39)	0.004	0.62 (0.40;0.84)	< 0.0001	0.39 (0.22;0.56)	< 0.0001

*EDSS* expanded disability status scale, *CI* confidence interval, *POMS* pediatric onset MS, *AOMS* adult onset MS, LOMS late onset MS

*The estimates were adjusted for gender, previous relapses, time from disease onset to first prescription, time spent on treatment and time from onset to first visit

The differences in LOMS vs POMS were more pronounced. The two curves diverged from the beginning of the follow-up (Year 1: delta_2_-EDSS 0.29 (0.12; 0.46), p = 0.0008) and reached a difference of the estimated EDSS score of 0.62 (0.40; 0.84, p < 0.0001) at year 5 (Fig. 2 and Table 2**).** The curves of LOMS and AOMS also diverged from the beginning of the follow-up but of a lesser extent, being the Year 1 delta_2_-EDSS 0.21 (0.08;0.34, p = 0.002) and 0.39 (0.22;0.56, p < 0.0001) at Year 5.

In 1037 patients (27.46% of the total cohort) a first 48-week CDA event occurred. PIRA events accounted for the 81.2% of the first CDA events (n = 842) in the whole population, for the 77.94% (n = 53) in POMS, for the 81.00% (n = 725) in AOMS and for the 86.49% (n = 64) in LOMS.

The median age (IQR) at the first PIRA event was 18.70 (17.00–19.40) years in POMS; 34.30 (28.50–41.40) years in AOMS; 56.50 (53.90–59.70) years in LOMS (P < 0.0001). The median (IQR) time to first PIRA event from the disease onset was 2.41 (1.28–3.30) years in POMS; 2.08 (1.36–3.10) years in AOMS and 2.17 (1.29–3.06) years in LOMS (p = 0.79).

**Fig. 3:**
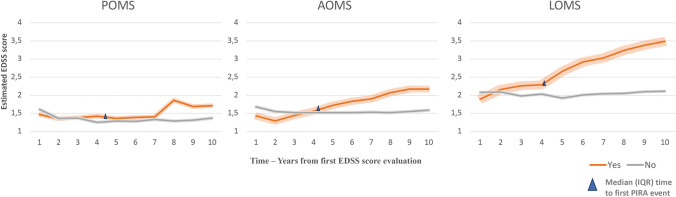
Curves of EDSS trajectories (95% CIs) over time stratified by PIRA

The curves of EDSS trajectories over time stratified by PIRA are reported in Fig. 3 and Table 3.

**Table 3:** Comparison of annual estimated mean delta_3_-EDSS scores (vs baseline) between patients who presented and who did not present a first PIRA event in POMS, AOMS and LOMS

Follow-up, years	POMS		AOMS		LOMS	
	Estimated mean Delta_3_-EDSS scores (95% CI) vs baseline	P value	Estimated mean Delta_3_-EDSS scores (95% CI) vs baseline	P value	Estimated mean Delta_3_-EDSS scores (95% CI) vs baseline	P value
1 year from first PIRA event	0.24 (− 0.03;0.51)	0.0822	0.57 (0.50;0.64)	** < .0001**	1.10 (0.84;1.36)	** < .0001**
2 years from first PIRA event	0.71 (0.44;0.98)	** < .0001**	0.80 (0.73;0.87)	** < .0001**	1.38 (1.12;1.64)	** < .0001**
3 years from first PIRA event	0.48 (0.20;0.76)	**0.0008**	0.83 (0.76;0.90)	** < .0001**	1.57 (1.31;1.83)	** < .0001**

In AOMS and LOMS groups, patients with PIRA showed a significantly (p < 0.0001) steeper increase in EDSS scores than those without PIRA, and this was evident from the first year after the occurrence of PIRA. In AOMS delta_3_-EDSS ranged from 0.57 (0.50; 0.64) at the first year to 0.80 (0.73; 0.87) at the second year and to 0.83 (0.76; 0.90) at the third year, and from 1.10 (0.84; 1.36) at the first year to 1.38 (1.12; 1.64) at the second year and to 1.57 (1.31; 1.83) at the third year in LOMS.

In the POMS group, patients who presented a PIRA event had also a significantly (p < 0.0001) steeper increase in EDSS scores than those without PIRA, but, unlike AOMS and LOMS, the two disability trajectories began to diverge later, two years after the first PIRA event, with delta3-EDSS ranging from 0.71 (0.44; 0.98) at the second year to 0.48 (0.20; 0.76) at the third year.

## Discussion

The complex and highly heterogeneous course of MS is underpinned by multiple pathogenetic mechanisms including inflammation, neurodegeneration, and patient-specific factors [4] which track different disability trajectories across individuals and over time.

Age is one of the major patient-specific factors contributing to making the disability trajectories different throughout time. A wide range of processes in peripheral immune cells and CNS cells, like astrocytes and microglia, change with aging and may affect pathophysiology, disability level, and treatment response in MS, highlighting the link between the clinical course of the disease and chronological age [21]. Accordingly, in this real-world study, applying LMMRM, which enabled us to compare the rate of the disability accrual among patients with different age at clinical onset, we provided evidence that the evolution of disability accrual is entirely different in POMS, AOMS, and LOMS regardless of treatment. No differences, indeed, were found among the groups in terms of total exposure time, proportion of follow-up time spent on pDMT and proportion of patients starting with a moderate efficacy or with a high efficacy DMT.

POMS, despite a higher frequency of relapses in the period between disease onset and the first visit, exhibited a slower disability accumulation in comparison to AOMS and LOMS.

Patients with PIRA showed a significantly steeper increase in EDSS scores than those without PIRA, in all age at clinical onset groups, as already reported in a previous paper [14]. Most importantly, we demonstrated that, although the median time to the first PIRA event from disease onset did not differ in POMS, AOMS and LOMS (2.41; 2.08 and 2.17 years, respectively), in POMS, the difference in yearly rates of delta_3_-EDSS increase over time between individuals with and without a PIRA is delayed and less pronounced than in AOMS and LOMS.

This finding is consistent with previous research that demonstrates children with MS not only recover from relapses considerably better than AOMS, but also considerably improve their functional system and EDSS scores three to five times more frequently than in adults [22]. The mechanisms of this improved recovery may be related to a greater capacity of remyelination and neuroplasticity in younger individuals, subsequently decreasing with aging [23, 24]. Disability trajectory in AOMS patients showed, after an initial reduction in the mean EDSS, a slight but constant increase in neurological disability. Delta-EDSS trajectory in LOMS showed a faster trend of progression compared to POMS and AOMS patients, and subjects with PIRA have a higher slope of disability compared to those not presenting it.

Several previous studies have highlighted the influence of age on disease course [25–29]. A recent study used group-based trajectory models to define four MS severity profiles among RRMS patients in the I-MS&RD, resulting in LOMS being associated with a rapid worsening of EDSS scores [30]. In line with these results, MS literature has shown that adults with disease onset at older age reach ambulatory disability milestones faster than younger adults [31].

Disability trajectories of MS patients have been already assessed in previous studies using data from the Big Multiple Sclerosis Data (BMSD) network [32, 33]. Three distinct disability trajectories have been observed in the more recent research on the long-term disability of people with secondary progressive MS, which was supposed to reflect different pathogenic processes of progression [33].

The observation of a slower accumulation of disability in treated POMS patients vs treated AOMS and LOMS patients during a 5-year follow-up period could support also the idea that treatment benefits on disability progression are highest in younger individuals and decrease with age. A meta-analysis of the main RCTs showed that the efficacy of DMTs decreases with increasing age [34]. Furthermore, a recent observational study by the I-MS&RD has shown that the efficacy of DMTs in delaying the achievement of EDSS 4.0 is greater in POMS and AOMS than in LOMS [35]. To reinforce the concept, another real-world study using data from the I-MS&RD revealed that in POMS the risk of persistent disability has decreased by 50–70% in recent diagnosis epochs, probably due to the improvement and timing in therapeutic and diagnosis [36].

A recent study from our group pointed out that PIRA can occur at any age and also in POMS, which is not protective against progression phenomena, rising dramatically its frequency with increasing age, in parallel with a worsening trend of disability [13]. Therefore, although PIRA resulted rarely detectable in children, POMS is currently not considered a protective factor against PIRA, which occurs throughout the disease course and gradually becomes more frequent with aging.

Some limitations of our study deserve discussion. Our analysis of disability accumulation relies only on the EDSS score. Although the baseline MRI features are a crucial prognostic factor, we could not include MRI data because of the lack of a systematic MRI acquisition and protocol analysis. Despite these considerations, our study used the large real-world database of the I-MS&RD, constantly improving quality of data and able to delineate disease evolution over time [37]. In conclusion, our results further support the new view of MS as a single, continuous process over time. We confirm that age at clinical onset remains highly relevant in determining the rate of disability accrual in contemporary cohorts of MS patients treated with DMTs. Moreover, the results showed PIRA occurrence in POMS is not uncommon, but its effect on the yearly rates of EDSS increase over time is delayed and less pronounced than in AOMS and LOMS.

## Disclosure

The authors report no conflicts of interest with respect to the contents of the current study, but note that the patients in the study were treated with a number of disease-modifying drugs and that authors have received advisory board membership, speakers honoraria, travel support, research grants, consulting fees or clinical trial support from the manufacturers of those drugs, including Actelion, Allergan, Almirall, Alexion, Amgen/Horizon, Bayer Schering, Biogen, Celgene, Excemed, Genzyme, Forward Pharma, Ipsen, Janssen/Johnson&Johnson, Medday, Merck Serono, Merz, Mylan, Novartis, Sanofi, Roche, Teva, BMS Cellgene and their local affiliates.

## Data Availability

Anonymized data, not published in the article, will be shared on reasonable request from a qualified investigator.
